# Contaminant release history identification in 2-D heterogeneous aquifers through a minimum relative entropy approach

**DOI:** 10.1186/s40064-015-1465-x

**Published:** 2015-10-31

**Authors:** Fausto Cupola, Maria Giovanna Tanda, Andrea Zanini

**Affiliations:** Department of Civil, Environmental, Land Management Engineering and Architecture, University of Parma, Parco Area delle Scienze, 181/A, 43124 Parma, Italy

**Keywords:** Minimum relative entropy, Transfer function, Pollutant transport, Inverse problems, Groundwater

## Abstract

The minimum relative entropy (MRE) method has been applied in a wide variety of fields since it was first introduced. Woodbury and Ulrych (Water Resour Res 29(8): 2847–2860, 1993, Water Resour Res 32(9): 2671–2681, 1996) adopted and improved this method to solve linear inverse problems in aquifers. In this work, the MRE method was improved to detect the source release history in 2-D aquifer characterized by a non-uniform flow-field. The approach was tested on two cases: a 2-D homogeneous conductivity field and a heterogeneous one (the hydraulic conductivity presents three orders of magnitude in terms of variability). In the latter case the transfer function cannot be described with an analytical formulation, thus, the transfer functions were estimated by means of a numerical procedure. In order to analyze the method performance in different conditions, two datasets have been used: observations collected at the same time at 20 different monitoring points, and observations collected at 2 monitoring points at several times. The observed data have been processed with and without a random error and the Boxcar and Gaussian probability distribution functions were considered as a priori information. The agreement between the true and the estimated data has been evaluated through the calculation of the normalized Root Mean Square error. The approach was able to recover the release history even in the most difficult case.

## Background

The interest in approaches that allow the estimation of pollutant source release in groundwater has increased exponentially over the last decades. This is due to the large number of groundwater reclamation procedures that have been carried out and the related costs that can be shared among the different actors if the release history is known. Moreover a reliable release history can be a useful tool for predicting the plume evolution, its concentration and the potential natural attenuation.

Several methodologies were developed in the ‘90 s to estimate the release history of a pollutant source for simple cases (1-D homogeneous aquifer) through a maximum likelihood (Wagner 1992), the Tikhonov regularization (Skaggs and Kabala 1994), a minimum relative entropy method (Woodbury and Ulrych 1993, 1996), and a geostatistical approach (Bagtzoglou et al. 1991, 1992; Snodgrass and Kitanidis 1997). All these methods recover the release history of a pollutant at a certain source location starting from the observation of several concentrations in the groundwater at a specific sampling time *T*.

The principle of minimum relative entropy (MRE) was introduced by Kullback (1959) and was used in several different fields (from geophysics to information theory (Woodbury and Ulrych 1996)). The MRE approach, with the aim of recovering the contaminant release history in aquifers, was applied on an analytical 1-D case (Woodbury and Ulrych 1996) and extended (Woodbury et al. 1998; Ulrych and Woodbury 2003) to a three-dimensional plume evolution described through an analytical solution. The MRE methodology was also compared to other approaches with the aim of highlighting the pros and cons (Kabala and Skaggs 1998; Neupauer et al. 2000; Neupauer and Borchers 2001; Woodbury 2011).

Due to the linearity of the governing differential equations of the transport problem, the afore mentioned methods adopt the convolution integral approach (Jury and Roth, 1990) to solve the advection–dispersion equation; then, the concentration at a time and at a point of the domain can be computed by means of the convolution of the mass release history at the source location with the transfer function (TF) that describes the effect in time, at a certain location of the aquifer, of an impulse release of a pollutant at the source. The TF can be analytically determined if the flow field is very simple (Skaggs and Kabala 1994; Woodbury and Ulrych 1996; Snodgrass and Kitanidis 1997; Butera and Tanda 2003), but in many practical applications, the characteristics of the groundwater flow field (conditioned on local heterogeneities, pumping wells, complex boundary conditions, etc.) do not allow an analytical formulation of the TF (Butera et al. 2006, 2013). At this aim, numerical procedures to compute the TF have to be developed (Neupauer et al. 2000; Michalak and Kitanidis 2004; Butera et al. 2006; Sun et al., 2006); among these the stepwise input function (SIF) procedure, introduced by Butera et al. (2006, 2013), has been adopted in the present study. The SIF procedure was successfully applied to compute the TFs considering 2-D synthetic homogeneous and heterogeneous aquifers (Butera et al. 2006, 2013), using data obtained from a laboratory device (Cupola et al. 2015) and using field data (Gzyl et al. 2014).

The objectives of the present work are to:

1. Extend the application of the MRE approach to contaminant release identification, developed by Woodbury and Ulrych (1993, 1996), to 2-D heterogeneous aquifers using as observations several concentration values at different observation points at a given time;
2. Extend the application of the MRE approach to contaminant release identification to heterogeneous multidimensional aquifers with availability of concentration values in few observation points at several monitoring times;
3. Analyze the performance of the MRE method, applied to contaminant release estimation, varying the prior information.

Before analyzing the heterogeneous cases we have verified the procedure by investigating case studies in a 2-D aquifer characterized by homogeneous hydraulic conductivity and uniform flow field, and in this paper we report the results of the application of the proposed method in four case studies: two homogeneous (HO1 and HO2) and two heterogeneous synthetic aquifers (HE1 and HE2). The results provide an evaluation of the influence of the heterogeneities on the solution of the inverse problem.

## Mathematical statements

### Groundwater transport

Equation (1) describes the transport process in an aquifer corresponding to the injection of a non-sorbing, non-reactive solute in a point source (Bear and Verruijt 1987):

1 $$\phi \frac{{\partial \left( {C\left( {{\mathbf{x}},t} \right)} \right)}}{\partial t} = \nabla \cdot \left[ {\phi {\mathbf{D}}\left( {{\mathbf{x}},t} \right)\nabla C\left( {{\mathbf{x}},t} \right)} \right] - \nabla \cdot \left[ {\phi {\mathbf{u}}\left( {{\mathbf{x}},t} \right)C\left( {{\mathbf{x}},t} \right)} \right] + s\left( {{\mathbf{x}}_{{\mathbf{0}}} ,t} \right)\delta ({\mathbf{x}} - {\mathbf{x}}_{{\mathbf{0}}} )$$

where *φ* [−] is the effective porosity (taken as spatially variable, but constant in time), **u**(**x**,*t*) [LT^−1^] is the effective velocity at location **x** and time *t* [T], **D**(**x**,*t*) [L^2^T^−1^] the dispersion tensor, *δ* the Dirac delta function, *C*(**x***,t*) [ML^−3^] the concentration at location **x** and time *t*, and

2 $$s\left( {{\mathbf{x}}_{0} ,t} \right) = Q_{in} \left( {{\mathbf{x}}_{0} ,t} \right)C_{in} \left( {{\mathbf{x}}_{0} ,t} \right)$$

where *s*(**x**_**0**_,*t*) [MT^−1^] is the amount of pollutant per time unit injected into the aquifer through the source located at **x**_**0**_, *Q*_*in*_(**x**_**0**_,*t*) [L^3^T^−1^] is the injection flow rate and *C*_*in*_(**x**_**0**_,*t*) [ML^−3^] is the concentration injected at **x**_**0**_ at time *t* [T].

The solution of Eq. (1), by considering uniform porosity, when associated with the initial and boundary conditions *C*(**x**,0) = 0; *C*(∞,t) = 0, is given by the following integral (Jury and Roth 1990):

3 $$C\left( {{\mathbf{x}},t} \right) = \int\limits_{0}^{t} {s\left( {{\mathbf{x}}_{0} ,\tau } \right)} f\left( {{\mathbf{x}},t - \tau } \right)d\tau$$

where *f(***x***, t*−*τ)* [L^−p^] is the TF that describes the effect at **x** at time *t* [T] by an impulse injection occurring at **x**_0_ at time *t*−*τ* (*p* is the dimension of the problem).

### Minimum relative entropy theory

The core of the method is the MRE inversion developed by Woodbury and Ulrych (1993, 1996), which is briefly summarized in the following.

Considering *p*(**y**) as a priori estimation of *q*(**y**), which is the multivariate probability distribution function (pdf) of occurrence of event $({\bf y}= {y_{1}}, \ldots, {y_{j}} \ldots {y_{N}}),$ the entropy of *q*(**y**) relative *p*(**y**) can be calculated as

4 $$E\left( {q,p} \right) = \;\int {q({\mathbf{y}})} \text{ln}\left[ {\frac{{q({\mathbf{y}})}}{{p({\mathbf{y}})}}} \right]d{\mathbf{y}}$$

The goal is to calculate the posterior pdf *q*(**y**) considering:

5 $$\int {q({\mathbf{y}})} d{\mathbf{y}} = 1$$

where the integration is over the full domain of the random variable **y** and the information is given in the form of expected value

6 $$\int {q({\mathbf{y}})} w_{i} ({\mathbf{y}})d{\mathbf{y}} = \overline{w}_{i}$$

where *w*_*i*_(**y**) and $\bar{w}_{i}$ are known and the index *i* goes from 1 to *M* which represents the number of known data (in this case is the number of observations).

So, after the minimization of the expression (4), the posterior estimate *q*(**y**) assumes the form (for more details about mathematical statement, see Woodbury and Ulrych 1993)

7 $$q({\mathbf{y}}) = p({\mathbf{y}})\exp \left[ { - 1 - \mu - \sum\limits_{i = 1}^{M} {\lambda_{i} w_{i} ({\mathbf{y}})} } \right]$$

where *μ* and *λ*_*i*_ are Lagrange multipliers determined by Eqs. (5) and (6). Therefore, the calculation of the multipliers is essential to estimate the posterior pdf.

### Inverse problem

For the case of a non-sorbing, non-reactive solute, the relationship between the concentrations observed at monitoring points and the release history is linear, as described in Eq. (3), and can be written as

8 $${\mathbf{z}} = \,{\mathbf{Hs}}$$

where **z** (*M* × 1) is an *M* known vector of observations, **s** = (*s*_1_,…*s*_*j*_,…*s*_*N*_) is the (*N* × 1) vector of unknowns, and **H** (*M* × *N*) is the transfer matrix which describes the relation between the unknowns and observations. It is defined as

9 $${\mathbf{H}} = \Delta t\left[ {\begin{array}{*{20}c} {f\left( {{\mathbf{x}}_{1} ,T - \Delta t} \right)} & \cdots & {f\left( {{\mathbf{x}}_{1} ,T - j\Delta t} \right)} & \cdots & {f\left( {{\mathbf{x}}_{1} ,T - N\Delta t} \right)} \\ \vdots & {} & \vdots & {} & \vdots \\ {f\left( {{\mathbf{x}}_{i} ,T - \Delta t} \right)} & \cdots & {f\left( {{\mathbf{x}}_{i} ,T - j\Delta t} \right)} & \cdots & {f\left( {{\mathbf{x}}_{i} ,T - N\Delta t} \right)} \\ \vdots & {} & \vdots & {} & \vdots \\ {f\left( {{\mathbf{x}}_{M} ,T - \Delta t} \right)} & \cdots & {f\left( {{\mathbf{x}}_{M} ,T - j\Delta t} \right)} & \cdots & {f\left( {{\mathbf{x}}_{M} ,T - N\Delta t} \right)} \\ \end{array} } \right]$$

where **x**_*i*_ denotes *i*-th monitoring point location, *T* [T] is the latest time considered (for instance the sampling time) and $\Delta t$ [T] is the time interval. The goal is to obtain an estimate ${\hat{\mathbf{s}}}$ (*N* × 1) of **s** that satisfies Eq. (8). Equation (8) can also be written in discretization form

10 $$z_{i} = \sum\limits_{j = 1}^{N} {f_{ij} } \cdot s_{j}$$

where *j* = 1,…, *N* is the index of the *j*-th unknown and *f*_*ij*_ is the shortened form of $f\left( {{\mathbf{x}}_{i} ,T - j\Delta t} \right)$. Let ${\hat{\mathbf{s}}}$ be the expected value of the random vector **s**; it can be calculated as: ${\hat{\mathbf{s}}} = \int\nolimits_{M} {\mathbf{s}} {\kern 1pt} q({\mathbf{s}})d{\mathbf{s}}$; consequently the Eq. (10) becomes

11 $$z_{i} = \int\limits_{M} {q({\mathbf{s}})} \left[ {\sum\limits_{j = 1}^{N} {f_{ij} s_{j} } } \right]d{\mathbf{s}}$$

where *q*(**s**) is the pdf of **s** and the integration is over all the allowed values of **s**. Equation (11) can be rewritten in the form of Eq. (6), where $z_{i}$ corresponds to the known observation $\bar{w}_{i}$ and $\sum\nolimits_{j = 1}^{N} {f_{ij} \cdot s_{j} }$ corresponds to *w*_*i*_(**y**). Woodbury and Ulrych (1996) constrained the value of **s** in the range (**L**, **U**), which represent the lower and upper bounds. This knowledge a priori is used to define a joint Boxcar pdf (uniform distribution between an upper and lower bound). The Boxcar pdf, *b*(**s**), is defined as

12 $$\left\{ {\begin{array}{ll} {b(s_{j} ) = \frac{1}{{U_{j} - L_{j} }},\quad L_{j} \le s_{j} \le U_{j} } \\ {b(s_{j} ) = 0,\quad {\text{otherwise}}} \\ \end{array} } \right.$$

where *L*_*j*_ and *U*_*j*_ are the individual lower and upper bounds. In this work the lower and upper bounds are considered constant for each *j* and in particular the lower bound is zero.

Let **r** be the expected value vector of the vector **s** which has *p*(**s**) as prior pdf, chosen in such a way that it has minimum relative entropy to a Boxcar pdf and let take that it assumes the expected value constraints, ${\bar{\mathbf{r}}} = (\bar{r}_{1} , \ldots ,\bar{r}_{j} , \ldots ,\bar{r}_{N} )$. Woodbury and Ulrych (1993) showed that the a priori estimation *p*(**s**) has the form

13 $$p({\mathbf{s}}){\kern 1pt} = {\kern 1pt} \prod\limits_{j = 1}^{N} {\frac{{ - \beta_{j} }}{{\exp \left( { - \beta_{j} U_{j} } \right) - 1}}\exp \left( { - \beta_{j} \bar{r}_{j} } \right)}$$

which is a multivariate truncated exponential; $\beta_{j}$ are Lagrange multipliers which can be estimated from the upper and lower bounds and the expected value constraints. By definition, *p*(**s**) satisfies the expected value constraints

$$\int {p({\mathbf{s}})} {\mathbf{s}}d{\mathbf{s}} = {\bar{\mathbf{r}}}$$

Finally, the posterior pdf *q*(**s**) is determined by minimizing its entropy relative to *p*(**s**) (subjected to the constraints of Eqs. (5) and (11). As demonstrated by Woodbury and Ulrych (1993), the posterior pdf has expression:

14 $$q({\mathbf{s}}){\kern 1pt} = {\kern 1pt} \prod\limits_{j = 1}^{N} {\frac{{ - a_{j} }}{{\exp \left( { - a_{j} U_{j} } \right) - 1}}\exp {\kern 1pt} \left[ { - s_{j} a_{j} } \right]}$$

where

15 $$a_{j} = \beta_{j} + \sum\limits_{i = 1}^{M} {\lambda_{i} f_{ij} }$$

in which ${\varvec{\uplambda}}$ is a Lagrange multipliers vector (*M* × 1). The expected value of the vector of unknowns is given by

16 $$\hat{s}_{j} {\kern 1pt} \left( {\varvec{\uplambda}} \right) = {\kern 1pt} \frac{{\exp \left( { - a_{j} U_{j} } \right) \cdot a_{j} U_{j} + \exp \left( { - a_{j} U_{j} } \right) - 1}}{{a_{j} \left[ {\exp \left( { - a_{j} U_{j} } \right) - 1} \right]}}$$

Substituting Eq. (16) into Eq. (10), we obtain

17 $$\hat{z}_{i} = \sum\limits_{j = 1}^{N} {f_{ij} } \hat{s}_{j} \left( {\varvec{\uplambda}} \right)$$

where $\hat{z}_{i}$ are the estimated concentrations.

Minimizing the objective function

18 $$F\left( {\varvec{\uplambda}} \right)_{i} = z_{i} - \hat{z}_{i} = z_{i} - \sum\limits_{j = 1}^{N} {f_{ij} \hat{s}_{j} ({\varvec{\uplambda}})}$$

the appropriate multipliers ${\varvec{\uplambda}}$ can be determined using the Newton–Raphson algorithm (Johnson 1987) (see Woodbury and Ulrych (1996), for more details).

### Determination of confidence intervals

As expressed by Eq. (14), the posterior pdf *q*(**s**) is non-Gaussian and confidence intervals cannot be easily derived in the classic way. However, they can be calculated starting from the cumulative distribution function (cdf) for **s**, defined as

$$\int\limits_{0}^{{\mathbf{s}}} {p({\mathbf{x}})\,d{\mathbf{x}} = P({\mathbf{s}})}$$

which, integrating terms by terms, gives (see for more details Woodbury and Ulrych 1993)

$$P(s_{j} ) = \frac{{\exp \left( { - a_{j} s_{j} } \right) - 1}}{{\exp \left( { - a_{j} U_{j} } \right) - 1}}\quad 0 \le s_{j} \le U\;,\;\forall \,j = 1, \ldots ,N$$

The goal is to define *s*_*j*_ corresponding to the defined probability *P*:

$$s_{j} = - \frac{{\text{log}\left[ {P\left( {{\text{exp}}\left( { - a_{j} U_{j} } \right) - 1} \right) + 1} \right]}}{{a_{j} }}$$

Assuming for instance *P* = 0.95, *s*_*j*_ results:

19 $$s_{j} = - \frac{{{\text{log}}\left[ {0.95\left( {\text{exp}\left( { - a_{j} U_{j} } \right) - 1} \right) + 1} \right]}}{{a_{j} }}$$

### Numerical TF computation

The SIF procedure developed by Butera et al. (2006) is a numerical strategy for the TF computation. According to Jury and Roth (1990), the concentration $C\left( {{\mathbf{x}},t} \right)$ at a certain location **x** at a time *t*, due to a release $s\left( {{\mathbf{x}}_{0} ,t} \right)$ at **x**_0_ at time *t* can be calculated through Eq. (3). If we assume a stepwise input function $s\left( {{\mathbf{x}}_{0} ,t} \right) = S_{0} H_{SF} \left( t \right) = Q_{0} C_{0} H_{SF} \left( t \right)$ [MT^−1^], where *H*_*SF*_(*t*) [−] is the Heaviside function, *C*_0_ [ML^−3^] is the concentration (known and constant in time), and *Q*_0_ [L^3^T^−1^] is the known and constant injected discharge, integral (3) becomes:

20 $$C\left( {{\mathbf{x}},t} \right) = \int\limits_{0}^{t} {Q_{0} C_{0} f\left( {{\mathbf{x}},t - \tau } \right)d\tau } = Q_{0} C_{0} \int\limits_{0}^{t} {f\left( {{\mathbf{x}},t - \tau } \right)d\tau } ,\quad t > 0$$

Taking the time derivative of Eq. (20) one gets

21 $$f\left( {{\mathbf{x}},t} \right) = \frac{1}{{S_{0} }}\frac{{\partial C\left( {{\mathbf{x}},t} \right)}}{\partial t},\quad t > 0$$

Equation (21) shows that the TFs can be computed at a generic point **x** by processing the concentration history at the same location originated by a step tracer injection at **x**_**0**_ and *t* = 0. The RHS of Eq. (21) can easily be obtained with a numerical flow and transport model able to simulate the effect of a pollutant injection into the aquifer of interest. The function *C*(**x**,*t*) is the so called breakthrough curve computed by the model at each monitoring point; applying the Eq. (21) for each monitoring point it is possible to fill the **H** matrix introduced in Eq. (9).

## Study cases

**Fig. 1:**
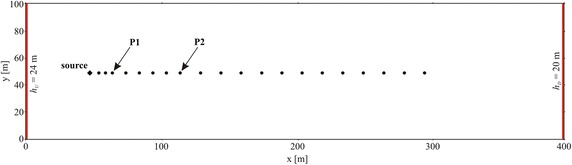
Sketch of the numerical model. The constant head boundary conditions are showed as *red lines*. The *black dots* indicate the measurement points of Case HO1, while P1 and P2 are the measurement points of Case HO2. The *black diamond* denotes the source location

The method proposed by Woodbury and Ulrych (1996) for a 1-D uniform flow field was extended to 2-D uniform and non-uniform flow fields. Both cases are based on numerical flow and transport models. The flow model, developed with MODFLOW (Harbaugh et al. 2000), reproduces a 2-D confined aquifer (1 layer) having rectangular shape (400 m long, 100 m wide) and 10 m thickness (Fig. 1). The computational grid is discretized into 2 × 2 × 10 m cells, obtaining 200 × 50 computational nodes. The boundary conditions are no flow at the North and South borders, constant head on the upstream (West) side *h*_*U*_ = 24 m and on the downstream (East) side *h*_*D*_ = 20 m (see Fig. 1). The hydraulic conductivity of the case (HO) is equal to 2.31 × 10^−4^ m/s, and the resulting flow through the aquifer is 2.3 × 10^−3^ m^3^/s. The transport model, developed with MT3D (Zheng and Wang 1999), uncoupled from the flow model, considers a solute non-sorbing, non-reactive release located in a point source at **x**_0_ (*x* = 49.0 m and *y* = 49.0 m, see Fig. 1). The longitudinal and transversal dispersivities and the porosity are assumed constant and equal to *α*_*L*_ = 1.0 m, *α*_*T*_ = 0.1 m, and *φ* = 0.20. The pollutant release at **x**_0_ is simulated as an injection with a constant water discharge and variable tracer concentration over time. The amount of the conservative pollutant per time unit injected into the aquifer at the source is given by Eq. (2), where $Q_{in} \left( {{\mathbf{x}}_{0} ,t} \right)$ [L^3^T^−1^] is the constant water discharge and $C_{in} \left( {{\mathbf{x}}_{0} ,t} \right)$ [ML^−3^] is the concentration release history, variable in time, of the injected solution. Following the works of Skaggs and Kabala (1994), Woodbury and Ulrych (1996), Snodgrass and Kitanidis (1997), Neupauer and Borchers (2001) and Butera et al. (2013), we considered a concentration release history with the expression:

22 $$C_{in} \left( {{\mathbf{x}}_{0} ,t} \right) = {\text{exp}}\left( {\frac{{ - \left( {\frac{t}{\Delta t} - 130} \right)^{2} }}{50}} \right) + 0.3\, \text{exp}\left( {\frac{{ - \left( {\frac{t}{\Delta t} - 150} \right)^{2} }}{200}} \right) + 0.5\, \text{exp}\left( {\frac{{ - \left( {\frac{t}{\Delta t} - 190} \right)^{2} }}{98}} \right)$$

Since $Q_{in} \left( {{\mathbf{x}}_{0} ,t} \right)$ is of unit value, the identification of the release history $s\left( {{\mathbf{x}}_{0} ,t} \right)$ is equivalent to the identification of the concentration history $C_{in} \left( {{\mathbf{x}}_{0} ,t} \right)$. The total time of the test is *T* = 600 days and the release history is discretized in 300 intervals with a time step of Δ*t* = 2 days. According to previous works the results are made dimensionless dividing the concentration by *C*_0_ = 1 mg/L and the time by the time step Δ*t*.

Two scenarios were studied: the first (HO1) recovered the release history by means of the observations collected at 20 monitoring points (black dots in Fig. 1) at time *T,* while the second (HO2) recovered the release history by means of the observations collected at only 2 monitoring points (P1 with coordinates 65.0 m, 49.0 m and P2 with coordinates 115.5 m, 49.0 m in Fig. 1) at 15 sampling times (see Table 1 for the summary of the study cases).

**Table 1:** Summary of the study cases and numerical results

Case	Hydraulic conductivity field	No. of monitoring points	Sampling times	No. of observations	Measurement error σ_R_ (mg/L)	nRMSE (%)		nRMSE (%)	
						Released history		Observed/Estimated	
						Expected function estimate		Expected function estimate	
						Gaussian	Boxcar	Gaussian	Boxcar
H01	Homogeneous	20 (Fig. 1)	*T* = 300Δ*t*	20	–	1.85	1.20	1.40	1.09
					10^−3^	2.20	1.34	1.52	1.16
H02	Homogeneous	2 (P1 and P2 of Fig. 1)	15 collected every 20Δ*t* up to *T*	30	–	5.37	5.72	2.43	2.10
					10^−3^	6.12	7.01	3.42	2.95
HE1	Heterogeneous	20 (Fig. 3)	*T* = 300Δ*t*	20	–	7.49	34.91	2.07	5.22
					10^−3^	9.06	38.72	4.38	6.71
HE2	Heterogeneous	2 (P3 and P4 of Fig. 3)	25 collected every 12Δ*t* up to *T*	50	–	4.59	4.88	4.57	3.84
					10^−3^	4.78	5.34	4.87	4.30

**Fig. 2:**
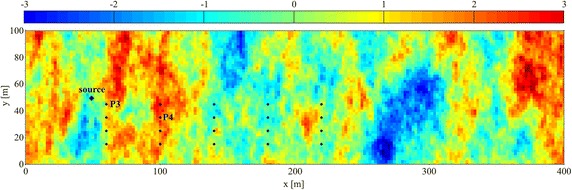
Normalized log-conductivity field (σ_*Y*_^2^ = 1.32). The *black dots* indicate the measurement points of Cases HE1; P3 and P4 are the measurements points of Case HE2. The *black diamond* is the source location

The non-uniform flow field (HE) was realized considering the heterogeneous hydraulic conductivity field proposed by Butera et al. (2013). The conductivity field was built considering an exponential covariance function and it is characterized by a mean value of 3.2 × 10^−4^ m/s, a standard deviation of 4.2 × 10^−4^ m/s and a correlation length equal to 20 m. The hydraulic conductivity has a very broad range from 2.6 × 10^−6^ m/s to 5.5 × 10^−3^ m/s. Figure 2 shows the normalized log conductivity field *Z* = (*Y* − *μ*_*Y*_)*/σ*_*Y*_ where *Y* = log *K* with mean *μ*_*Y*_ and standard deviation *σ*_*Y*_. The log conductivity field variance is *σ*_*Y*_^*2*^ = 1.32 and the resulting flow through the aquifer is about 1.20 × 10^−3^ m^3^/s. The assumption of a known hydraulic conductivity field is rather unrealistic; in fact, in field condition it is difficult to obtain detailed information on hydraulic parameters and for this reason there is a huge collection of literature on estimating hydraulic conductivity variability (for example see Fienen et al. 2009; Zanini and Kitanidis 2009; Cardiff et al. 2013). The present paper has the goal of testing the goodness of the MRE procedure applied to contaminant release history identification, assuming known the conductivity field and transport parameters.

**Fig. 3:**
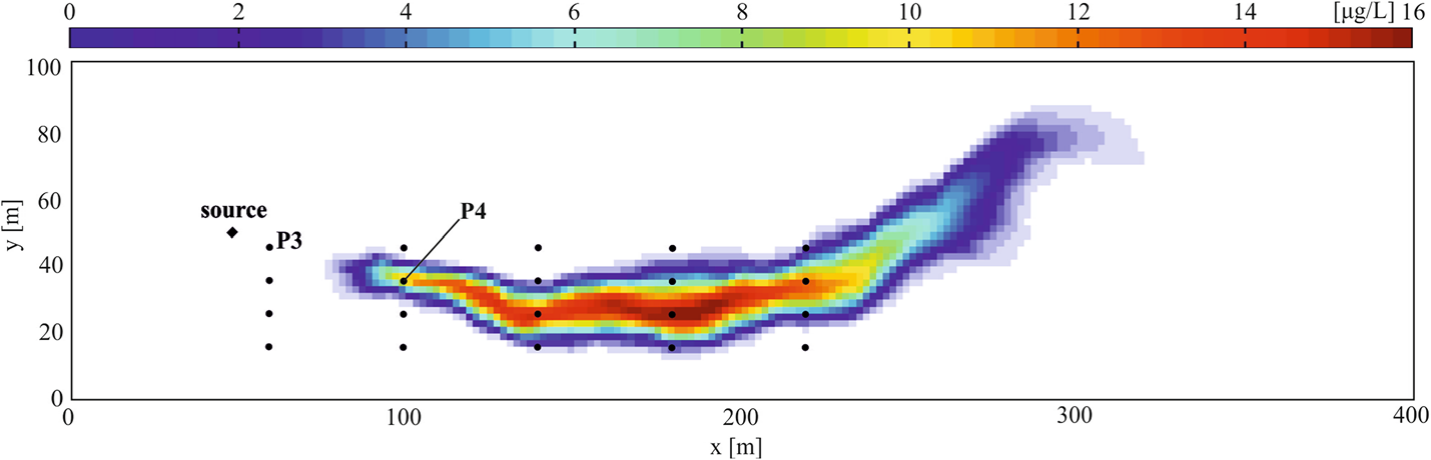
Plume at *T* = 300Δ*t.* The *black dots* indicate the measurement points of Cases HE1; P3 and P4 are the measurements points of Case HE2. The *black diamond* is the source location

The transport parameters and the source location are the same as the previous cases HO1 and HO2. As for the HO case, two scenarios were studied: in the first case (HE1) the release history is recovered using the observations from 20 monitoring points (black dots in Fig. 2) at time *T*, while in the second application (HE2) the observations from 2 monitoring points (P3 with coordinates 60.0 m, 45.0 m and P4 with coordinates 100.0 m, 35.0 m in Fig. 2) with 25 samples at different times have been used. The Fig. 3 shows the concentration distribution due to the heterogeneities at time *T*.

The tests were carried out in the following sequence: (1) the numerical flow and transport models were used to estimate the TFs at each monitoring points by using the SIF procedure; (2) the numerical models were used to compute the concentrations at the monitoring points due to the release function described by Eq. (22); (3) the observations collected at the monitoring points were used in the inverse procedure aimed at recovering the release history at the source location.

The MRE approach requires information on the prior pdf distribution of the release history; in this work we tested the method under the two alternatives of a Gaussian (Eq. 23) and a Boxcar (Eq. 24) function.

23 $$\bar{r}\left( t \right) = \alpha_{g} \cdot e^{{ - \frac{{\left( {t - \mu_{g} } \right)^{2} }}{{2 \cdot \sigma_{g}^{2} }}}} \quad \forall t \in \left[ {0,T} \right]$$

24 $$\left\{ \begin{aligned} \bar{r}\left( t \right) = \bar{r}_{b,{\rm max} } \quad t_{s} \le t \le t_{e} \hfill \\ 0\quad {\text{elsewhere}} \hfill \\ \end{aligned} \right.\quad t_{s} ,t_{e} \in \left[ {0,T} \right]$$

where $\alpha_{g}$, $\mu_{g}$, $\sigma_{g}^{2}$ are the parameters of the Gaussian function, $\bar{r}_{b,\rm{max} }$ is the constant value used as expected value constraint, and *t*_*s*_ and *t*_*e*_ are the temporal limits of the Boxcar function. The parameters of the prior information, then, were different in each case: using the Gaussian expression (23) one has to assume mean and variance value while the function (24) requires an estimate of the start and end of the injection and its maximum value. Finally, also the upper values of the **U** (Eq. 12) have to be estimated.

Besides the graphical comparison, the results were analyzed considering the normalized root mean square error (nRMSE) between the computed and observed concentrations and between the estimated and true release history (Table 1).

### Case HO1: analysis with concentration data collected at 20 locations at the same time

**Fig. 4:**
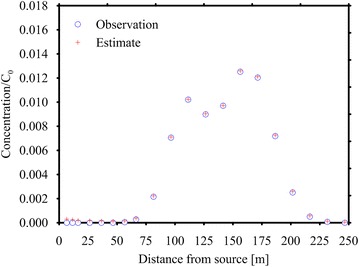
Observed and estimated concentrations at time *T* for Case HO1. These data were collected at 20 monitoring points depicted in Fig. 1. The estimated concentrations shown here were calculated by using the Gaussian function as prior estimate. The observed concentrations are corrupted by error

**Fig. 5:**
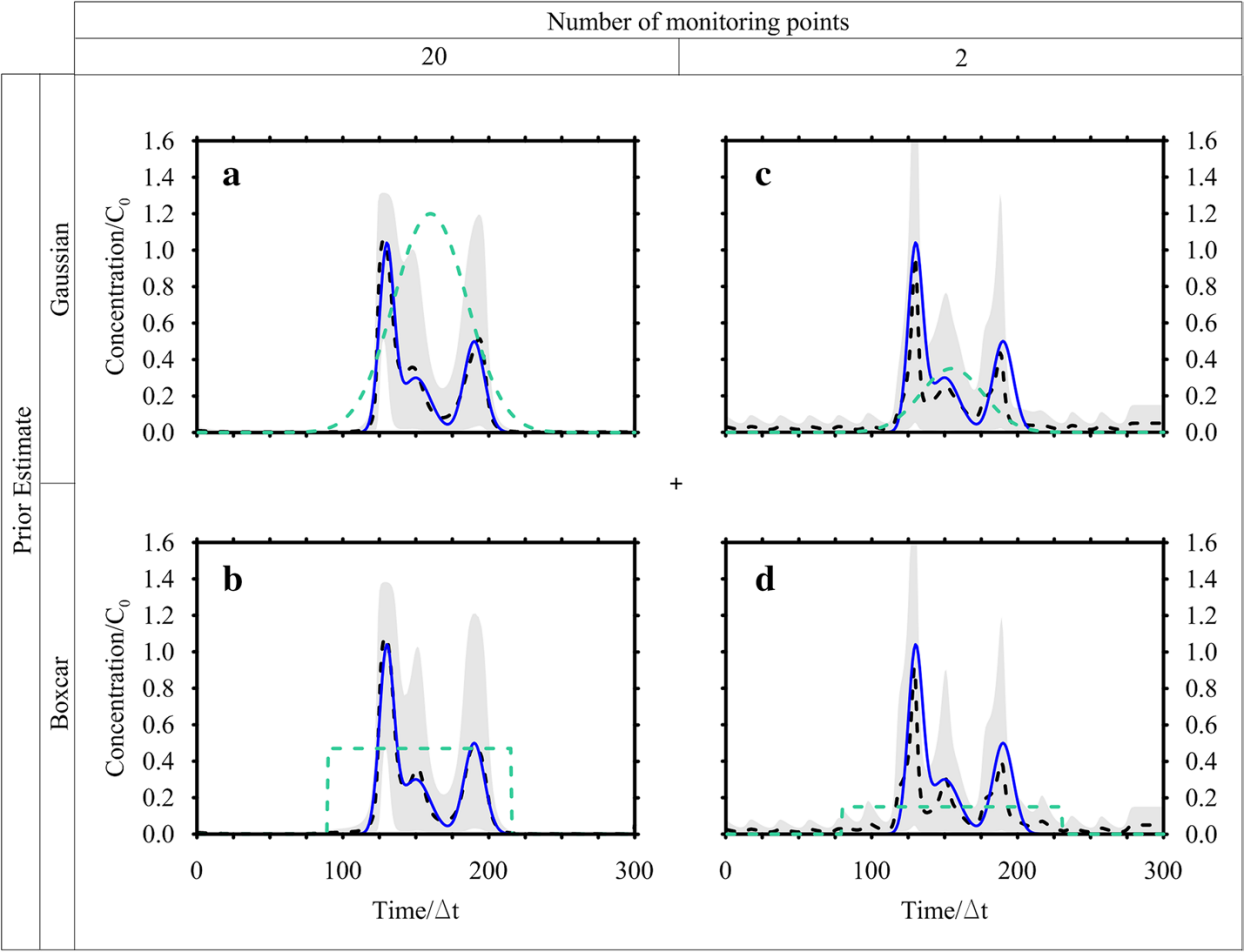
Recovered release function using observations corrupted by error. True solution (*blue line*), best estimate (*dashed line*), prior estimate (*green dashed line*) and 5–95 % confidence interval (*grey band*). **a** Case HO1 with Gaussian function estimate; **b** Case HO1 with Boxcar function estimate; **c** Case HO2 with Gaussian estimate; **d** Case HO2 with Boxcar estimate

20 observations collected at 20 positions (Fig. 1) at time *T* = 300Δ*t* were considered in the recovery process (Fig. 4) in order to determine the release history at 300 time intervals (unknowns), i.e. the **s** vector with dimension (300 × 1). The above described (23) and (24) expressions for the expected value of the prior distribution of the release history have been considered; the results obtained are shown in Fig. 5a, b. Initially, the processed data were considered error free, and then they were corrupted with a random error with mean value equal to 10^−3^ mg/L; in Figs. 4 and 5 the results obtained using corrupted observations are shown.

The results of the procedure, using a Gaussian function as expected value (Fig. 5a), are very satisfactory. The recovered release history is very similar to the true one. Moreover, the concentrations estimated at the monitoring point (due to the recovered release history) are very close to the one observed (Fig. 4). The maximum nRMSE computed between the recovered and true release history results 2.20 % (see Table 1), while the one maximum computed between the estimated and observed concentration at the monitoring points is 1.52 %. Considering a Boxcar function as expected value (Fig. 5b), the estimated release history is even better than the previous one; that is confirmed by the nRMSE computed, in the worst case, between the recovered and true release history in 1.34 % (see Table 1), while the one computed between the estimated and observed concentration at the monitoring points is 1.16 %.

The results are very sensitive to the prior information required by the method, in particular to the upper value **U** and to the parameters of the expected value function. The knowledge of these parameters requires a rough idea of the true release history, so the goodness of the results is conditioned by some strong, in a certain sense arbitrary, hypothesis. Several tests, not reported here for briefness, were carried out to investigate the sensitivity of the MRE method to the expected value functions and to the upper value. Basically, the results showed that the solutions are relatively insensitive to the kind of previously expected value functions, while it is more sensitive to the upper value. In particular, if the maximum of the expected value function is greater than the upper value, the method is not able to converge to a solution. Moreover, the results worsen as the value (assumed constant) of the upper bound **U** increases.

### Case HO2: analysis with concentration data collected at 2 locations at different times

**Fig. 6:**
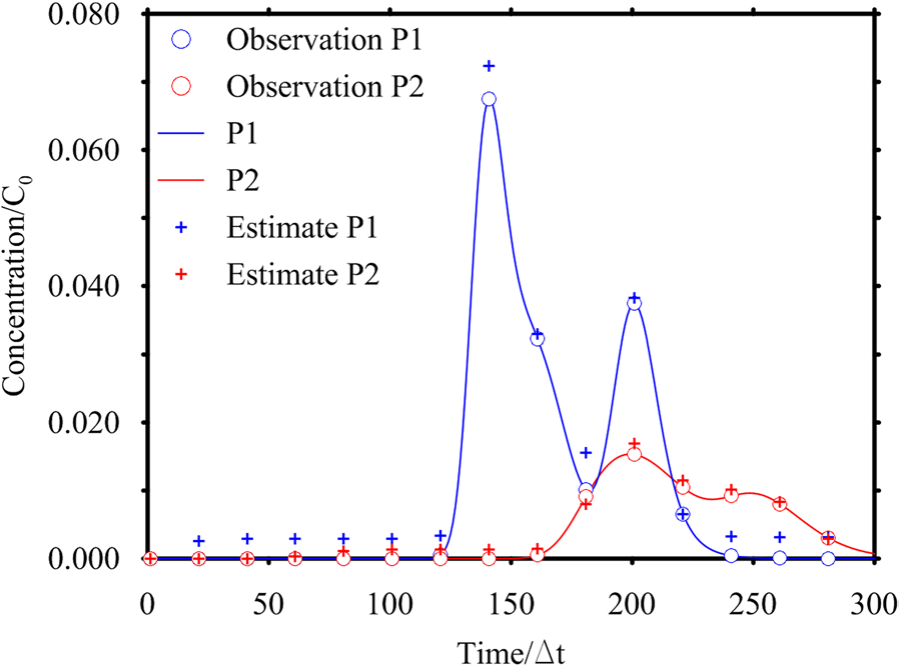
Observed and estimated concentrations for Case HO2. The line represents the concentration at the monitoring point. The circle represents the sampled concentration and the cross is the estimated concentration by using the Gaussian function as expected estimate value. The observed concentrations are corrupted by error

The goal of this second application is to investigate the reliability of the MRE method using several pieces of information collected at few locations at different times. This case is very realistic, as it is common to have only few monitoring points and several sampled concentration values for each point at different times. The test case is the same as case HO1, but only two observation points (P1 and P2 of Fig. 1) were used. At these points 15 concentration values are considered available in 600 days, with a time step equal to 20Δ*t* (Fig. 6). The transfer functions for the two observation points were the same as the previous case but the **H** matrix of Eq. (9) becomes $${\mathbf{H}} = \Delta t\left[ {\begin{array}{*{20}c} {{\mathbf{H}}_{P1} } & {{\mathbf{H}}_{P2} } \\ \end{array} } \right]^{T}$$ where **H**_*Pi*_ are the matrices that contains the TF of each monitoring point evaluated at the specified sampling time; the vector of the observations can be written as: $${\mathbf{z}} = \left[ {\begin{array}{*{20}c} {{\mathbf{z}}_{P1} } & {{\mathbf{z}}_{P2} } \\ \end{array} } \right]^{T}$$ so that Eq. (8) is still valid.

Figure 5c, d show the results once again using two different expected value functions (Gaussian and Boxcar). In these cases the results do not appear to be as good as the previous case; in particular neither of the release histories recovered is totally included in the 5–95 % confidence interval, although both the peaks values and times are well estimated. By considering the nRMSE (Table 1) it is clear that the results are less accurate than the ones obtained in case HO1.

### Case HE1: analysis with concentration data collected at 20 locations at the same time

The tests were carried out in the sequence described above for the cases HO1 and HO2. In these cases (HE1 and HE2), the monitoring points cannot be the same as the ones used in the cases HO1 and HO2, since the plume evolution is very different: in fact the local heterogeneities increase the complexity of the recovering process originating a plume evolution that involves the monitoring points of Fig. 1 to a negligible extent.

**Fig. 7:**
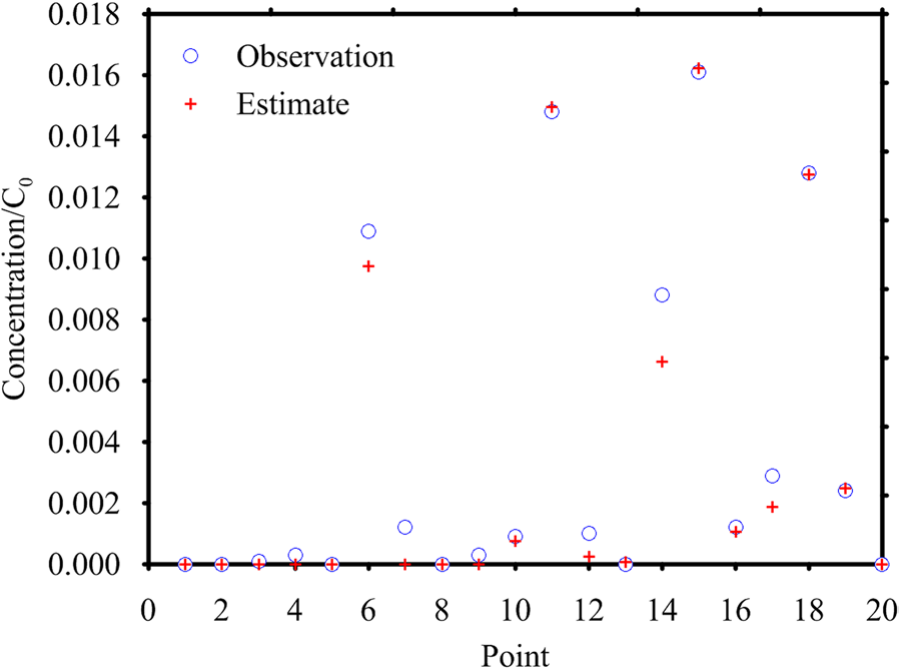
Observed and estimated concentrations at time *T* for Case HE1. These data were collected at 20 monitoring points depicted in Fig. 3. The estimated concentrations shown here were calculated by using the Gaussian function as expected estimate value. The observed concentrations are corrupted by error

**Fig. 8:**
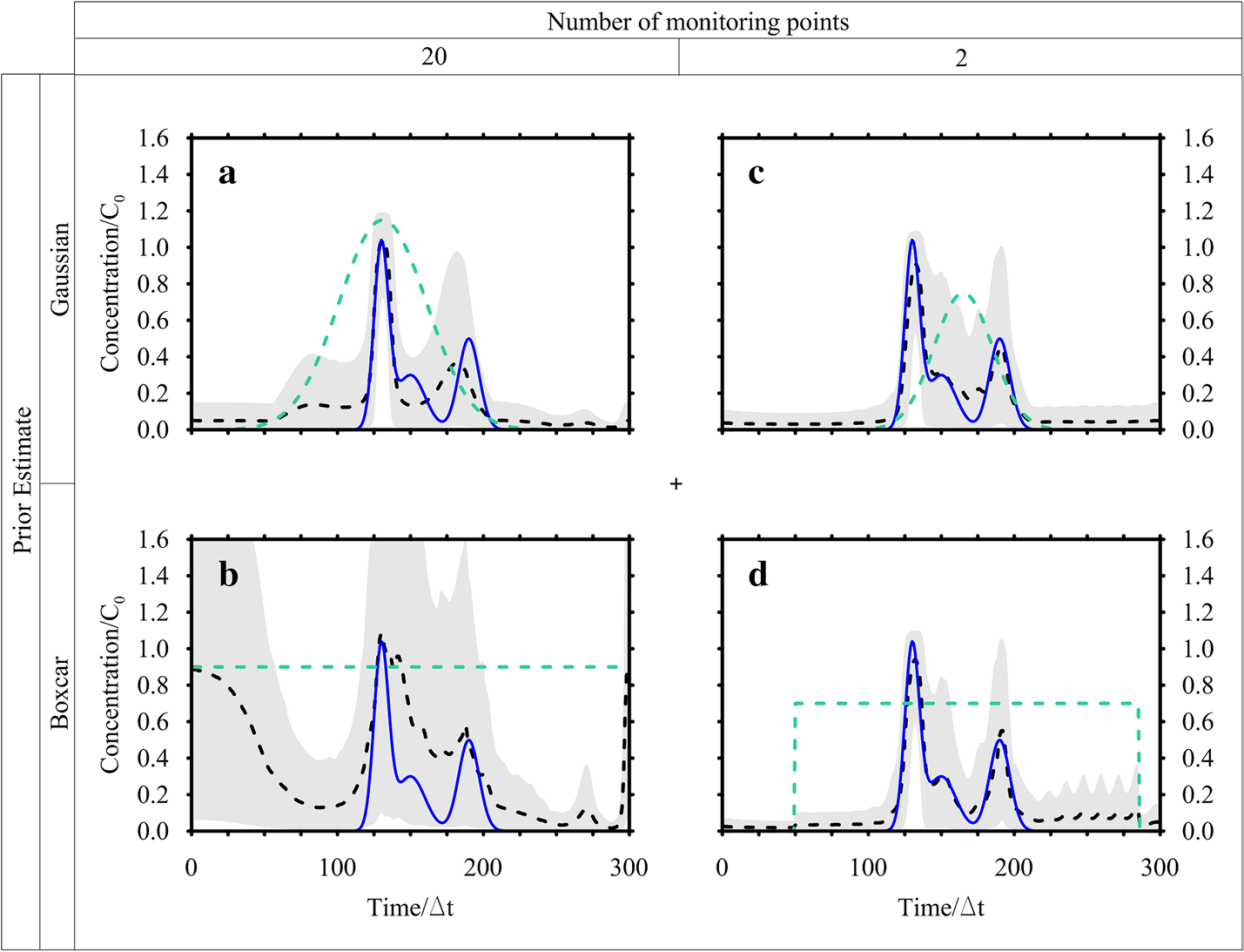
Recovered release function using observations corrupted by error. True solution (*blue line*), best estimate (*dashed line*), expected value function (*green dashed line*) and 5–95 % confidence interval (*grey band*). **a** Case HE1 with Gaussian function estimate; **b** Case HE1 with Boxcar function estimate; **c** Case HE2 with Gaussian function estimate; **d** Case HE2 with Boxcar function estimate

The monitoring points, depicted in Fig. 3, are located in the nodes of a grid covering the region 60 ≤ *x* ≤ 220 m and 15 ≤ *y* ≤ 45 m. After the calculation of the TFs at each point, the MRE method has been applied at first without considering any measurement error, with good results: then the observations have been corrupted by a random error normally distributed with σ_*R*_ = 10^−3^ mg/L. Figure 7 shows the observations (corrupted by errors) collected at *T* = 600 days in each monitoring point and those estimated through the inverse procedure. Two prior expected functions have been considered, the Gaussian and the Boxcar expression. The results are shown in Fig. 8a, b and Table 1. Considering the Gaussian function (Fig. 8a) the MRE procedure estimates the true release history reasonably for both with and without error cases (see Fig. 8; Table 1). The nRMSE, computed between the true and the estimated (with the Gaussian prior pdf and in presence of measurement errors) release history is close to 9 %. While, analyzing the release history obtained through the boxcar expression (Fig. 8b), even if it is overestimated, it seems acceptable for *t*/Δ*t* > 100 only and this misfit is quantified in an nRMSE of about 39 %. The nRMSE between the computed and observed concentrations shows, in all cases, values below 7 % that indicates an acceptable estimation of the observations.

### Case HE2: analysis with concentration data collected at 2 locations at different times

**Fig. 9:**
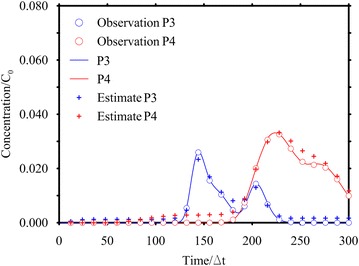
Observed and estimated concentrations for Case HE2. The *line* represents the concentration at the monitoring point. The *circle* represents the sampled concentration and the cross is the estimated concentration by using the Gaussian function as expected estimate value. The observed concentrations are corrupted by error

In this scenario, only two monitoring points (P3 and P4 depicted in Fig. 3) are considered and 25 concentration values are taken as observations in 600 days, with a time step equal to 12Δ*t* (Fig. 9).

Like in HE1, two different conditions have been considered: the first assumes noise-free data, while the second considers an error with a standard deviation equal to 10^−3^ mg/L. The processing of the error free data has provided very encouraging results, with nRMSE values between the computed and observed data and the true and estimated release histories close to 4 %. In presence of measurement errors, both the Gaussian (Fig. 8c) and Boxcar functions (Fig. 8d) have resulted in good release histories (nRMSE about 5 %) and in a satisfactory reproduction of the observed concentrations (see Fig. 9 for the Gaussian expression and Table 1).

Both graphical (Figs. 8, 9) and numerical (Table 1) results show that, in this scenario, the release history is better recovered and presents a smaller confidence interval than in HE1; surely this is due to the high values of the TF at the monitoring points P3 and P4, which provide very reliable information. Moreover, 50 observations (as a total) are available instead of only 20 (HE1). It is important to remark that considering information in few points at several times allows to capture the leading and the trailing edge of the plume and provides information about the entire release history. Whereas considering information at several monitoring points at a specific time does not allow to capture the leading edge of the plume and consequently the data do not contains information about the early times of the release history distribution. That demonstrates the need for either good spatial coverage or good temporal coverage or both.

### Comparison to other approach

Butera et al. (2006, 2013) have presented an improvement of the geostatistical approach to contaminant release history identification developed by Snodgrass and Kitanidis (1997) and have tested the method on several cases. The Case HE1, discussed in this paper, is the same of Case 1 presented by Butera et al. (2013); this allows a comparison of the results between the two methodologies. Figure 8a, b show the estimated release histories using two different prior estimates. It is evident that the Gaussian prior provides better results than the Boxcar one. Comparing Figs. 5a to 8 of Butera et al. (2013), it seems that both methods recover well the two main peaks of the release function and do not identify the middle one. The MRE reproduces better the magnitude and the timing of the peaks, but on the downside it presents non zero values on the first part of the release. This problem seems reduced in the geostatistical approach but instead a larger confidence interval is present.

The Case HE2 (Figs. 8c, d) can be compared to the results obtained in Case 3 (true source location in Fig. 7) of Butera et al. (2013). The geostatistical approach estimates a large confidence interval at the head and at the tail of the estimated release function; while MRE at the same times estimates non zero concentration values but it presents a narrow confidence interval.

However, both methods are able to reproduce the three peaks of the release history, their magnitude and timing that in the reclamation and forensic activities are the most important issues (Atmadja and Bagtzoglou, 2001).

## Discussion and conclusions

In this paper the minimum relative entropy method for recovering the contaminant release history in 2-D homogeneous and heterogeneous aquifers involved in a pollutant event has been applied. In the applications here referred, the SIF numerical method (Butera et al., 2006) has been applied for the computation of the TFs. The performances of the MRE have been tested in uniform and non-uniform flow field, considering different scenarios, and with different prior information. They have been quantified by the nRMSE calculated between the true source release and the estimated ones, and between the concentration observed and the computed ones; the nRMSE quantities are summarized in Table 1.

Two scenarios have been considered: the first uses 20 observations collected at different locations at the same time while the second processes several observations collected at different times at two monitoring points only. For each scenario, the method has been applied using two different expected value functions ${\bar{\mathbf{r}}}$: the Gaussian and the Boxcar expression. The results show that in the homogeneous case (HO) the methodology works very well; this is clearly shown by the nRMSE, which is lower than 2.2 % in all tests. The release function is well recovered in the two cases studied with the two different expected value functions. It is important to remark that in this case the release function presents 300 unknowns (*N*) and it is recovered with 20 observations (*M*) (HO1, with a ratio *M*/*N* of 0.067) or 30 observations (HO2, with a ratio *M*/*N* of 0.100), while Woodbury and Ulrych (1996) estimated 200 unknowns with 61 observations with a ratio *M*/*N* of 0.305. The results have demonstrated that the method is efficient with few observations too.

Regarding the non-uniform flow field, the presented cases have a heterogeneous hydraulic conductivity field with a log conductivity variance of σ_*Y*_^2^ = 1.32; the results obtained in these cases are not as good as in the homogeneous ones but still appreciable and meaningful. The method performs better using several observations collected in few monitoring points rather than one observation at different points. However, in HE1, 300 unknowns (N) were estimated by using 20 observations (ratio equal to 0.067), while in HE2 50 observations collected at two different points at different times (ratio equal to 0.167) have been used. The observations have been compared with the ones reproduced by the forward transport model by using the estimated release history as source term: the results show that the agreement is acceptable in both cases. Note again that the ratio of the number of observations on the unknowns (*M/N*) is very low (0.067) in comparison with the ratio *M*/*N* = 0.305 used by Woodbury and Ulrych (1996) in their applications to homogeneous 1-D aquifers. The present results have demonstrated that the method is efficient with few observations too and the less performance can be ascribed to the non-uniformity of the flow field rather than to the amount of available data.

Another important issue, in a non-uniform context, is the monitoring point location: it is crucial to have available information in spots with high peak TF values; in HE1, several monitoring points have TFs with a very low peak value which means that monitoring locations provide very poor information about the pollutant event development and numerically they generated a matrix **H** that is ill-conditioned. This **H** matrix causes failing in the convergence procedure and consequently errors on the results. During the design of a monitoring network we suggest to consider the monitoring points that present the higher peak values of the TF. It is important to note that a monitoring point can have a high peak TF value and a zero concentration; this information is very important and allows to bound the plume.

The comparison between the different cases shows that, while in the homogenous case the spatial data are more informative and the methodology performs better, in the heterogeneous case, the temporal data allow a better estimation of the release history. Basically, they give opposite indications. This is due to the position of the monitoring points respect the shape of the plume. In fact, in the case HO1 all the points are interested by the pollution phenomenon and all of them give information different from the others. All together, they provide a very good framework of the evolution of the plume. In case HO2, the information given by two points is not as comprehensive as those provided in case HO1. Moreover, since the field is homogenous, the concentration data at P1 and P2 cannot be so different (considering their position). Looking at the case HE1, instead, several monitoring points have TFs with a very low peak value. For sure, they provide information, but globally, the quantity of data is less than the case HO1.

In case HE2 the two monitoring points are along the plume and they provide very good information about the concentration evolution. This proves, once again, how much a good reconstruction of the flow field is important, in order to design a monitoring network.

It is worth to add some considerations about the prior information that the MRE method requires. If the assumed prior is very far from the right posterior and the TF is poor at the point or in time, the Newton–Raphson method cannot converge to a solution. The method requires a prior expected value of the unknown release history and a guess of the collocation in time. By using a Gaussian expression, this means to choose the mean and the variance of the Gaussian curve, while, for the Boxcar, a rough idea of the concentration maximum value and the start and the end time of the injection are necessary. The MRE prior should reflect what is known before the new information is considered and should be maximally uncommitted with respect to unknown information (Woodbury and Ulrych 1993; Woodbury et al. 1998). For this reason the prior information are not subjective to the user; a useful guidance on their selection was given in Woodbury and Ulrych (1996) and Woodbury (2011).

The MRE approach, thanks to the lower limit **L** (that in this work is set to zero), does not require the transformation of variables, as other methods do, to constrain the unknown function (for instance the concentrations) to only positive values. This fact avoids the increase of unknowns (parameters of the transformation) in the inverse procedure and does not affect the stability of the procedure that can happen choosing the wrong transformation.

In conclusion, the MRE approach developed by Woodbury and Ulrych (1993, 1996) integrated with the SIF procedure (Butera et al. 2006) works fine in simple study cases (such as 1-D and 2-D with uniform flow field), even with few observations, and in complex cases, the reliability of the results depends on the number of the available observations and the location of the monitoring points. However, the procedure is considered satisfactory; all three peak times have been detected, and the best estimate falls in the 5–95 % confidence interval for all cases. Thanks to the numerical modeling and the SIF procedure, the MRE can be applied to field cases, using the data that normally are spread in space and time.

Future studies will use the experimental data collected in laboratory (Cupola et al. 2015), where different transport processes can be realized in controlled conditions. Another interesting subject is to test the MRE procedure for the simultaneous identification of the source location and release history and compare the results with the ones obtained by Butera et al. (2013) by using a geostatistical approach.
